# Performance Results and Concentrations of Biochemical Indices and Mineral Elements in Blood Serum of Fatteners Fed Diets Containing Mixtures of Raw Seeds of Pea (*Pisum sativum* L.) or Blue Lupin (*Lupinus angustifolius* L.)

**DOI:** 10.3390/ani10050858

**Published:** 2020-05-15

**Authors:** Marcin Sońta, Martyna Batorska, Justyna Więcek, Anna Rekiel

**Affiliations:** Department of Animal Breeding, Institute of Animal Sciences, Warsaw University of Life Sciences—SGGW, Ciszewskiego 8, 02-786 Warsaw, Poland; marcin_sonta1@sggw.edu.pl (M.S.); justyna_wiecek@sggw.edu.pl (J.W.); anna_rekiel@sggw.edu.pl (A.R.)

**Keywords:** fatteners, alternative protein, pea seed, blue lupin seed, blood serum parameters

## Abstract

**Simple Summary:**

The aim of this study was to determine the effect of using pea and blue lupine seeds as replacers for genetically modified soybean meal (SBM-GM), traditionally used in pig feeding, on their production results, health and body homeostasis. The public is often opposed to the use of feed with GM materials in animal nutrition, which is why the search for alternative sources of feed protein that can be used in commercial production is underway. Despite the differences in the level of biochemical indices and mineral elements concentration in blood serum in pigs fed diets containing pea or blue lupine seeds, the study did not show their negative impact on the production parameters and body homeostasis. The results obtained indicate that legumes seeds—pea and blue lupin—can offer an alternative to SBM-GM. It has been shown that pea or blue lupine seeds in the diet of fattening pigs allows to achieve similar production results and do not adversely affect the homeostasis of the animal body.

**Abstract:**

Two experiments were conducted with fatteners (♀ (Landrace × Yorkshire) × ♂ duroc), 50 animals each (10 pigs per group). The fatteners from the control group (C) were administered feed mixtures with genetically modified soybean meal (SBM-GM) used as the only protein source; whereas these from experimental groups (E1–E4) received feed mixtures in which the SBM-GM was replaced with increasing amounts of raw seeds of pea (Experiment I) or blue lupin (Experiment II): E1—5.0%, E2—10.0%, E3—15.0%, and E4—17.5%. Once the fattening period was completed, production results were determined, and selected blood serum indices were assayed to establish the effect of the nutritional factor on body homeostasis and health status of the animals. Pigs from all groups revealed a similar growth rate and meatiness (*p* > 0.05). In Experiment I serum analyses showed lower (*p* < 0.001) concentrations of: cholesterol in E1, E3 and E4; creatinine in E1 and E4 and urea in E3 and E4, compared to the C. In Experiment II, lower (*p* < 0.001) concentrations of aspartate aminotransferase, alanine-aminotransferase, total protein, and Mg were determined in the serum of fatteners from E1–E4 compared to the C. Even though values of all analyzed blood markers differed among the groups, in most cases they fitted within reference values for the species, which indicates the maintenance of body homeostasis. Study results show that there are no contraindications to the use of pea and blue lupin seeds as alternative feed materials to SBM-GM in pig fattening.

## 1. Introduction

The primary source of protein in feed for monogastric animals (pigs, poultry) is genetically modified soybean meal (SBM-GM), an expensive and imported component. In the pig production, the aim is to reduce the cost of feed without compromising the production performance of pigs. The replacement of SBM-GM is possible using alternative protein sources such as pea seeds, lupins and rapeseed meal (RSM).

The usability of various plant-derived protein feed materials as partial replacers for SBM-GM has been confirmed in experiments conducted with model and farm animals [1], including pigs [2,3,4,5]. Even though the legume seeds contain some anti-nutrients, they represent a fine raw material for the production of feedstuffs and foodstuffs due to high contents of protein, including valuable amino acids, as well as vitamins and minerals [6,7]. As demonstrated by Sedlaková et al. [8], the legumes exert a positive effect on the energy balance, blood levels of glucose and lipids, arterial pressure, peristalsis and defecation. Amarowicz and Pegg [9] and also Wang and Clements [10] claimed them to be natural sources of antioxidants. Following a diet that is rich in legumes, which provide high contents of dietary fiber, saponins, phytosterols, and oligosaccharides and have a low glycemic index [11], and at the same time poor in saturated fats may allow controlling lipid homeostasis in the body.

The incorporation of white and blue lupin protein into a diet for rats has been shown to decrease concentrations of cholesterol and triglycerides in blood of experimental animals compared to the control ones [12], whereas the use of protein isolates and dietary fiber from lupins—to reduce blood cholesterol level by 21% [13]. In the study with an in vivo experimental model of diet-induced hypercholesterolemia, the use of a lupin protein hydrolysate combined or not with insoluble lupin fiber in a diet for rats decreased concentrations of triglycerides in their plasma and liver and had a positive impact on glucose metabolism [14]. In turn, lupin seeds inclusion into a diet for monogastric animals had a beneficial effect on cholesterol level; its decrease was demonstrated in chickens [15] and pigs [16].

Body homeostasis may fluctuate as a result of great effort during intense fattening. The use of raw legume seeds in rat feeding can upset the body homeostasis and diminish production results [17], because they are carriers of various anti-nutrient substances [18]. In pig nutrition, however, feed mixtures with yellow lupin seeds [16] did not reduce production results, but the blood levels of biochemical and mineral markers varied. Few authors only have examined the impact of legume seeds on biochemical blood indices of pigs [16,19,20,21,22]. Breeding work on legume plants is carried out toward reducing contents of anti-nutritional factors. The use of pea seeds and lupins with low contents of anti-nutritional substances in pig nutrition has no negative effect on their production results and their body homeostasis. Considering this, the present study was undertaken to determine the effect of various contributions of pea seeds and blue lupin seeds in feed mixtures for fatteners on their production results and on their biochemical and mineral blood markers, provided that the mixtures are balanced in terms of protein and energy levels.

## 2. Materials and Methods

According to the Polish law and the EU Directive (no. 2010/63/EU), the experiment did not require approval from the Local Ethical Committee as it was done by local farmers on a small scale (in the production conditions).

Pilot studies were carried out under the production conditions of the family pig producing farm. The farm uses available cereal components and a standard premix in pig nutrition. Two experiments were conducted with growing pigs administered feed mixtures in which SBM-GM was replaced by either pea seeds or blue lupin seeds.

### 2.1. Animals and Housing

The experiments were conducted with 3-breed weaners (♀ (Landrace × Yorkshire) × ♂ duroc), 50 pigs per experiment (at barrows: gilts ratio of 1:1). Prior to experimental fattening, the animals were weighed, ear-pierced and dewormed. Their average body weight before the beginning of Experiment I and II was 26.7 and 33.5 kg, respectively. The animals used in each experiment (n = 50) were randomly divided into 5 groups (control group—C and experimental groups—E1, E2, E3, E4), and placed in group pens, 10 animals each. The fattening was carried out in two stages, i.e., in Experiment I: Stage I—6 weeks and Stage II—7 weeks; and in Experiment II: Stage I—4 weeks and Stage II—6 weeks.

### 2.2. Nutrition

Fattening was performed using iso-energetic and iso-protein complete feed mixtures [23]. In the control group (C), SBM-GM served as the only source of protein, whereas in diets of fatteners from the experimental groups (E1–E4) the SBM-GM was replaced with increasing doses of raw pea seeds—Hubal variety (Experiment I) or blue lupin seeds—Regent variety (Experiment II), i.e., E1—5.0%, E2—10.0%, E3—15.0% and E4—17.5% (Table 1 and Table 2). The loose feed mixture and water were administered ad libitum. The feed mixtures used in the experiment were determined for their chemical composition [24].

### 2.3. Blood Analysis

All fatteners had 12 h of starvation before slaughter. After slaughter, the percentage of meat content in hot carcasses (meatiness) was determined and blood samples were collected. Blood samples were centrifuged (3500 rpm, 10 min) and serum obtained was stored at a temperature of −20 °C. Biochemical indices were determined with an Accent 200 analyzer (Cormay, Warsaw, Poland) using multicalibrators level 1, HP and HN sera and test reagents (Cormay, Warsaw, Poland). Determinations were carried out for concentrations of: alkaline phosphatase—ALP, aspartate aminotransferase—ASPAT, alanine aminotransferase—ALAT, albumins—ALB, total protein—TP, glucose—GLU, cholesterol—CHOL, triglycerides—TG, creatinine—CREA, urea—UREA, as well as calcium—Ca, phosphorus P, magnesium—Mg and iron—Fe.

### 2.4. Statistical Analysis

The results from experiments were subjected to statistical analysis using the IBM SPSS Statistics 25 package. Means and standard deviations were used to describe the data, without FCR. Differences between the groups were assessed using linear regression models with the control group used as a reference. Overall, significance of the model was obtained using F statistics, whereas significance for each level of the grouping variable was obtained using t statistics. *p* < 0.05 was considered to denote statistically significant differences.

## 3. Results

### 3.1. Production Results

**Figure 1:**
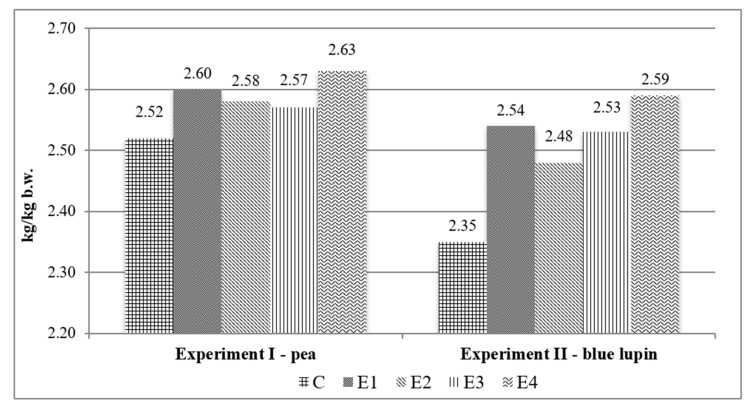
Feed conversion ratio (kg of feed mixture/kg of body weight gain) in the entire fattening period in Experiments I and II.

Experiment I. At the beginning of fattening, the body weight of fatteners from groups C, E1, E2, E3 and E4 was comparable and reached 26.7 ± 0.9 kg on average (Table 3). Body weight (BW) gains during fattening did not differ among groups. In the control group feed conversion ratio (FCR) was 2.52 kg feed/kg gain BW gain, for the fatteners from groups E1–E4 was higher by 0.05–0.11 kg feed/kg gain BW (Figure 1). Meatiness of the fatteners was above 60%, irrespective of group (Table 3).

Experiment II. The initial body weight of the fatteners was similar in all groups and reached 33.5 ± 1.5 kg on average (Table 3). Average daily body gain was very high and ranged from 1272 g in the E3 group to 1201 g in the E4 group, control pigs had achieved of 1260 g/day (Table 3). The FCR calculated for experimental groups vs. control group differed by 0.13–0.24 kg of feed mixture/kg of body weight gain (Figure 1). The highest meatiness was determined for the fatteners from group E3 and the lowest one—for these from group E4 (Table 3).

### 3.2. Biochemical and Mineral Blood Indices

Experiment I. No changes were demonstrated in concentrations of the biochemical (ALP, ASPAT, ALB, TP, GLU, TG) and mineral blood indices (Ca and Fe) among the experimental groups E1–E4 (Table 4). The lower concentrations—compared to the control group—were noted in E1 (*p* < 0.011), E3 (*p* < 0.001) and E4 (*p* < 0.008) groups for CHOL, in E1 (*p* < 0.001) and E4 (*p* < 0.002) groups for CREA, as well as in E3 (*p* < 0.023) and E4 (*p* < 0.001) groups for UREA.

Experiment II. Lower concentrations of ASPAT (*p* < 0.001), ALAT (*p* < 0.001), TP (*p* < 0.001) and Mg (*p* < 0.01) were determined in the serum of fatteners from experimental groups, compared to the control group (Table 5). The serum level of CREA and UREA was lower only in the pigs from groups E1, E2 and E3 vs. control group. The animals from group E4 had the highest serum levels of ALB, CHOL, UREA, Ca, P and Fe.

## 4. Discussion

In both experiments, very good production results were achieved by all fatteners regarding daily body weight gains, feed conversion ratio and meatiness. The growth rate and feed conversion ratio of growing pigs have significantly increased in recent years [25]. Improvement of pure-bred pigs of dam and sire breeds caused that the hybrid pigs usually achieve even higher production results than the pure-bred animals (heterosis effects). The high genetic potential of the pigs regarding their productive traits and the high-quality balanced feed mixtures with pea seeds (5.0%–17.5%) or blue lupin seeds (5.0%–17.5%) administered to them allowed achieving high production results in the present study.

The biochemical and hematological blood markers are indicative of the general body homeostasis [26]. Their determination and analysis are important as their values depend on multiple factors, including these related to the feeding system and feed quality, as confirmed in studies conducted by, i.e., Czech and Grela [19], Burek and Grela [27] and Czech et al [20].

The values of hematological, biochemical and mineral blood indices are determined by, i.e., species and breed [26,28], immune system excitation state [29], age [22,30,31,32], fattening season [33] and environmental conditions [34,35].

The values of biochemical indices and mineral elements concentration in blood serum determined in the present study varied and were statistically significantly different between group C and groups E1–E4. In our previous study [21], values of biochemical blood indices analyzed for the fatteners from the control group (feed mixture without yellow lupin) and from experimental groups (E1—7.5% and E2—15% of yellow lupin seeds in complete feed mixtures) showed some tendencies. A descending tendency was demonstrated for the following indices (group: C > E1 > E2): ALP, ASPAT, ALAT, TP, CHOL, HDL, CREA, UREA, as well as Ca and P. Serum levels of the other indices tested (ALB, GLU, TG, Mg, Fe) were lower in groups E1 and E2 vs. C. These tendencies were, however, not confirmed in the present study. As reported by Gajęcki [36], a decrease in TP, BUN and GLU below the reference values coupled with an increase in AST and ALAT levels and with high levels of total bilirubin and ALP were noted in pigs fed a diet deficient in protein and carbohydrates. A diet of this type makes tissue metabolism turn into catabolism. In the present study, the catabolic processes were not superior over the anabolic ones, which was indicated by very good daily body weight gains of the fatteners from all feeding groups in both experiments. These results may confirm that the feed mixtures with national protein components used were well-balanced and covered demands of growing pigs for nutrients at Stage I and II of fattening.

One of the indicators determined in Experiment I and II was total protein (TP). TP concentration as well as either an increase or decrease in its value provide information about protein supply from feed mixtures. From the nutritional point of view, an increased blood level of TP may be indicative of energy deficits. In Experiment I, its concentration in the serum of pigs from the experimental group was similar to control animals, which indicates a sufficient protein supply and adequate energy to protein ratio upon partial replacement of soybean meal with pea seeds in feed mixtures. In Experiment II, similar to the study conducted by Prandini et al. [37], the concentration of TP was significantly (*p* < 0.01) reduced in groups E1, E2 and E3 vs. C. However, the very good production results of animals in this experiment, including fatteners from groups E1–E4, points to the adequate coverage of nutritional demands of growing pigs for protein, when blue lupin seeds were used in feed mixtures as a replacer for SBM-GM.

Concentrations and changes in the concentrations of lipid indices, i.e., total cholesterol and triglycerides (TG), enable evaluating metabolic transformations in the body [38]. Cholesterol is a precursor of steroid hormones synthesis, therefore its concentration in the blood is positively correlated with feed intake and health status of animals. Triglycerides (TG) represent the major form in which lipid reserves are stored in the body; they are accumulated in adipocytes and released to the blood circulation when needed [38].

Alanine (ALAT) and aspartate (ASPAT) aminotransferases are intracellular cytosol enzymes. Damage of cells, hepatocytes in particular, leads to their leakage from damaged cells, which increases their level in blood plasma [39]. According to Šimek et al. [40], diet supplementation with white lupin seeds can have a positive impact on the life cycle of cells by protecting them against excess degradation, which was confirmed in the present study wherein blood levels of ASPAT and ALAT were shown to decrease (Experiment II).

Experiments with legume seeds inclusion into feed mixtures for monogastric animals (pigs and poultry) have demonstrated their hypocholesterolemic effect as one of their nutritional advantages [13,16,41,42]. This effect is most likely due to reduced cholesterol absorption from the gastrointestinal tract as a result of greater reabsorption of bile acids, which contributes to diminished solubility of cholesterol [16]. This effect was confirmed in the present study but only in Experiment I, i.e., upon SBM-GM replacement with pea seeds. In Experiment II, the experimental factor had no explicit effect on blood levels of lipid indices which were found to vary, e.g., the lowest concentration of CHOL was determined in the serum of pigs receiving diet with 5.0% of blue lupin seeds and the highest one—in the serum of fatteners fed a diet with 17.5% of blue lupin seeds. Considering literature data, after inclusion of blue lupin (10%) to feed ratios for experimental pigs, Zralý et al. [43] reported increased blood levels of TP, ALB, GLU, TG, CHOL, HDL and ASPAT as well as decreased levels of ALAT, ALP, Ca and P. Prandini et al. [37] demonstrated that diets containing pea and lupin seeds had no negative effect on liver functions, as concentrations of ALP, ASPAT and ALAT in blood of experimental pigs were similar to these determined in blood samples of the control animals.

The interpretation of values of the biochemical and mineral blood indices obtained in the present study is difficult in the light of literature data that may be treated as guidelines or standards [26,32,44] and in comparison to results demonstrated by various authors [13,16,20,21,41,42,43]. This difficulty lies in various pig genotypes, feed mixture compositions as well as different contributions of legumes in feed mixtures in the present experiment and in investigations reported by other authors.

A comparison of the values of biochemical indices and mineral elements concentration in blood serum determined in experiments I and II with reference values for the species provided by Friendship and Henry [44], Klem et al. [32] and Winnicka [26] allowed concluding that most of them fitted within the range of reference values, although some of them slightly diverged from these values. The values of selected biochemical and mineral blood indices matching the upper reference values for the domestic pig species or negligibly exceeding them could be attributed to the high growth potential of pigs used for fattening.

Mineral components serve many important functions and influence chemical reactions in animal bodies (growth, reproduction, lactation). Concentrations of minerals determined in blood of the fatteners in the present study are comparable with these reported by other authors [33,45]. The higher Ca concentration in blood plasma can be due to a higher concentration of albumins [46], which was however not observed in our study. According to Furcht [47], a decreased serum level of phosphorus may result from the intake of a diet poor in phosphorus (phosphorus-deficient dietary components like cereals or insufficient mineral supplementation). An additional cause can be a high calcium level which diminishes phosphorus availability; however, this was not confirmed in the present study. The concentration of Fe in serum is generally claimed to be a marker of inflammatory processes [48]. In our study, it fitted the reference values for the domestic pig species, which may be indicative of no inflammatory conditions in the experimental animals.

## 5. Conclusions

The values of biochemical indices and mineral elements concentration in blood serum determined in the study for the fatteners indicate maintenance of their body homeostasis. Study results show that there are no contraindications to the use of pea and blue lupin seeds in the amounts ranging from 5.0% to 17.5% as alternative feed materials to SBM-GM in pig fattening. These results are beneficial from the environment as well as the consumer point of view.

## Figures and Tables

**Table 1:** Contribution (%) of raw materials in feed mixtures administered at Stage I and II of fattening in Experiment I.

Raw Feed Materials	Groups				
	C	E1	E2	E3	E4
**I fattening stage**					
Barley	35.0	30.0	25.0	15.0	5.0
Triticale	24.0	19.2	20.3	22.1	28.3
Wheat	20.0	25.0	25.0	30.0	30.0
Oats	5.0	5.0	5.0	5.0	5.0
Soybean meal GM	13.0	9.7	8.3	6.4	2.0
Rapeseed meal	–	2.5	2.5	2.5	7.8
Pea seeds	–	5.0	10.0	15.0	17.5
Soybean oil	–	0.6	0.9	1.0	1.4
Premix *	3.0	3.0	3.0	3.0	3.0
**Analyzed nutritional value (%)**					
Dry matter	87.6	87.4	86.9	87.3	87.5
Crude protein	16.4	16.5	16.3	16.5	16.3
Ether extract	2.6	2.7	2.7	2.7	2.8
Crude fiber	3.9	3.9	4.0	4.1	4.1
Crude ash	3.9	4.0	3.9	4.1	3.9
**Calculated nutritional value (%)**					
Metabolic energy (MJ/kg)	13.22	13.21	13.21	13.21	13.21
Lysine	1.05	1.06	1.09	1.10	1.10
Methionine + cysteine	0.64	0.64	0.64	0.63	0.67
Threonine	0.70	0.71	0.71	0.71	0.72
Tryptophan	0.20	0.19	0.19	0.19	0.19
Calcium	0.81	0.82	0.82	0.82	0.86
Phosphorus	0.54	0.55	0.55	0.54	0.58
Sodium	0.17	0.19	0.21	0.23	0.24
**II fattening stage**					
Barley	35.0	25.0	15.0	10.0	8.2
Triticale	32.0	26.5	30.0	30.0	30.0
Wheat	10.0	21.6	24.7	30.0	30.0
Oats	10.0	10.0	10.0	6.9	5.0
Soybean meal GM	10.5	6.6	4.8	3.0	-
Rapeseed meal	–	2.5	2.5	2.2	6.0
Pea seeds	–	5.0	10.0	15.0	17.5
Soybean oil	–	0.3	0.5	0.4	0.8
Premix *	2.5	2.5	2.5	2.5	2.5
**Analyzed nutritional value (%)**					
Dry matter	86.8	86.4	87.0	86.7	86.1
Crude protein	15.3	15.4	15.5	15.2	15.1
Ether extract	2.5	2.6	2.6	2.6	2.7
Crude fiber	4.1	4.2	3.9	4.2	3.9
Crude ash	4.5	4.7	4.5	4.7	4.6
**Calculated nutritional value (%)**					
Metabolic energy (MJ/kg)	13.17	13.16	13.17	13.17	13.16
Lysine	0.94	0.94	0.95	0.96	0.97
Methionine + cysteine	0.60	0.61	0.61	0.60	0.61
Threonine	0.64	0.64	0.64	0.64	0.65
Tryptophan	0.18	0.18	0.18	0.17	0.17
Calcium	0.68	0.70	0.69	0.69	0.71
Phosphorus	0.51	0.51	0.51	0.50	0.52
Sodium	0.15	0.17	0.19	0.21	0.21

* Premix composition: lysine—12.10%; methionine—2.65%; threonine—5.05%; tryptophan—0.25%; calcium—20.50%; phosphorus—1.80%; sodium—5.00%; iron—4000 mg; manganese—2400 mg; zinc—2600 mg; copper—800 mg; iodine—55.0 mg; selenium—13.50 mg; vitamin A—260,000 IU; vitamin D3—69,000 IU; vitamin E—4 700 mg; vitamin K3—68 mg; vitamin B1—68 mg; vitamin B2—170 mg; vitamin B6—105 mg; vitamin B12—830 mcg; vitamin C—1000 mg; folic acid—27.00 mg; pantothenic acid—410 mg; niacinamide B3—690 mcg; biotin—3450 mg; choline chloride—10,000 mg; aroma, antioxidant: 1b (E320-BHA, E321-BHT, E324—ethoxyquin) 550 mg/kg; enzymes: 4a E-1 640 6—phytase (EC 3.1.3.2.6 n-5000 FTU/g) 17 500 FTU/kg, (E1600 endo 1,4-beta-xylanase, EC 3.2.1.8–22,000 VU/g; 425,000 VU/kg, endo 1,3 beta-glucanase EC 3.2.1.6–30,000 VU/g, 57,000 VU/kg); raw material composition: calcium carbonate, monocalcium phosphate, (monophosphate) sodium chloride 1.8.1.9, herbal mix 10 g/kg.

**Table 2:** Contribution (%) of raw materials in feed mixtures administered at Stage I and II of fattening in Experiment II.

Raw Feed Materials	Groups				
	C	E1	E2	E3	E4
**I fattening stage**					
Barley	36.6	12.0	11.0	5.4	2.3
Triticale	15.0	30.0	30.0	33.0	35.0
Wheat	25.0	33.7	35.0	35.0	33.0
Oats	5.0	3.0	–	–	–
Soybean meal GM	15.0	10.5	8.0	5.5	2.0
Rapeseed meal	–	2.5	2.5	2.5	6.0
Blue lupin seeds	–	5.0	10.0	15.0	17.5
Soybean oil	0.4	0.3	0.5	0.6	1.2
Premix *	3.0	3.0	3.0	3.0	3.0
**Analyzed nutritional value (%)**					
Dry matter	87.7	87.4	87.3	87.1	87.4
Crude protein	16.3	16.4	16.3	16.2	16.4
Ether extract	2.6	2.6	2.7	2.7	2.8
Crude fiber	3.9	3.9	4.2	4.2	4.1
Crude ash	3.9	4.1	4.1	4.1	3.9
**Calculated nutritional value (%)**					
Metabolic energy (MJ/kg)	13.23	13.19	13.20	13.21	13.20
Lysine	1.09	1.06	1.04	1.03	1.05
Methionine + cysteine	0.66	0.65	0.64	0.63	0.64
Threonine	0.72	0.71	0.70	0.70	0.71
Tryptophan	0.21	0.20	0.19	0.19	0.18
Calcium	0.87	0.89	0.89	0.89	0.91
Phosphorus	0.53	0.52	0.51	0.50	0.51
Sodium	0.17	0.18	0.18	0.18	0.18
**II fattening stage**					
Barley	36.7	12.0	11.4	8.8	5.8
Triticale	20.0	30.0	30.0	33.0	35.0
Wheat	20.0	32.3	35.0	35.0	33.0
Oats	8.0	7.5	3.0	–	–
Soybean meal GM	12.8	8.2	5.6	3.1	–
Rapeseed meal	–	2.5	2.5	2.5	5.6
Blue lupin seeds	–	5.0	10.0	15.0	17.5
Soybean oil	–	–	–	0.1	0.6
Premix *	2.5	2.5	2.5	2.5	2.5
**Analyzed nutritional value (%)**					
Dry matter	87.6	87.9	87.4	87.5	88.0
Crude protein	15.4	15.6	15.3	15.2	15.3
Ether extract	2.2	2.1	2.3	2.2	2.3
Crude fiber	3.9	3.8	4.2	4.0	4.1
Crude ash	4.0	3.9	4.0	4.1	4.3
**Calculated nutritional value (%)**					
Metabolic energy (MJ/kg)	13.20	13.19	13.19	13.19	13.20
Lysine	0.98	0.95	0.96	0.96	0.96
Methionine + cysteine	0.63	0.63	0.61	0.60	0.60
Threonine	0.66	0.65	0.65	0.64	0.65
Tryptophan	0.19	0.19	0.18	0.17	0.17
Calcium	0.74	0.75	0.76	0.76	0.78
Phosphorus	0.50	0.49	0.48	0.47	0.48
Sodium	0.14	0.15	0.15	0.15	0.16

* Premix composition—see: Table 1.

**Table 3:** Daily body weight gains and meatiness of fatteners (Experiment I and II) ($\bar{x}\;\pm \;$SD).

Specification	Groups ^1^					*p*-Value
	C	E1	E2	E3	E4	
	Experiment 1					
Initial body weight, kg	26.4 ± 1.1	27.1 ± 0.8	26.9 ± 0.9	26.5 ± 0.8	26.5 ± 1.0	0.336
Final body weight, kg	123.4 ± 9.6	123.0 ± 8.7	124.8 ± 7.5	122.1 ± 7.3	116.5 ± 9.7	0.255
Average daily body weight gain, g	1104 ± 119	1090 ± 97	1113 ± 87	1086 ± 80	1022 ± 108	0.294
Meatiness, %	60.0 ± 0.8	60.6 ± 1.9	60.1 ± 2.3	60.4 ± 2.0	59.7 ± 2.4	0.854
	**Experiment II**					
Initial body weight, kg	33.5 ± 1.4	33.1 ± 1.5	33.9 ± 1.5	33.7 ± 1.8	33.4 ± 1.5	0.810
Final body weight, kg	125.5 ± 8.7	123.5 ± 5.8	125.1 ± 4.9	126.6 ± 7.7	121.1 ± 7.4	0.452
Average daily body weight gain, g	1260 ± 118	1238 ± 82	1249 ± 70	1272 ± 107	1201 ± 92	0.529
Meatiness, %	58.9 ± 1.9	58.9 ± 2.9	58.9 ± 2.0	59.2 ± 2.5	58.1 ± 2.5	0.878

^1^ C—soybean meal; E1—5.0% exp. I—pea seeds or exp. II blue lupin seeds; E2—10.0% pea seeds or blue lupin seeds; E3—15.0% pea seeds or blue lupin seeds; E4—17.5% pea seeds or blue lupin seeds.

**Table 4:** Biochemical and mineral blood indices ($\bar{x}\;\pm \;$SD), Experiment I.

Specification	Units	Groups ^1^					*p*-Value
		C	E1	E2	E3	E4	
Alkaline phosphatase (ALP)	U/L	115 ± 17	121 ± 13	141 ± 28	126 ± 30	115 ± 12	0.166
Aspartate aminotransferase (ASPAT)	U/L	63.2 ± 17.0	78.3 ± 28.8	63.9 ± 19.5	61.5 ± 23.3	81.9 ± 27.8	0.342
Alanine-aminotransferase (ALAT)	U/L	46.8 ^a^ ± 7.5	44.6 ± 11.4	34.2 ^a^ ± 13.1	54.8 ± 11.7	48.4 ± 8.7	0.003
Albumin (ALB)	g/L	47.7 ± 3.1	43.5 ± 6.4	46.9 ± 6.4	43.1 ± 7.9	39.9 ± 8.8	0.129
Total protein (TP)	g/L	80.4 ± 4.9	71.1 ± 10.7	77.2 ± 9.2	66.8 ± 18.4	77.7 ± 20.0	0.199
Glucose (GLU)	mmol/L	8.9 ± 1.7	8.9 ± 1.1	7.9 ± 1.9	7.5 ± 1.4	8.5 ± 2.5	0.134
Cholesterol (CHOL)	mmol/L	3.1 ^A,b,a^ ± 0.4	2.5 ^a^ ± 0.4	2.8 ± 0.2	2.4 ^A^ ± 0.6	2.5 ^B^ ± 0.7	0.011
Triglycerides (TG)	mmol/L	0.8 ± 0.2	0.7 ± 0.1	0.7 ± 1.0	0.8 ± 0.3	0.8 ± 0.3	0.720
Creatinine (CREA)	μmol/L	161 ^A,B^ ± 12	117 ^A^ ± 22	141 ± 14	139 ± 19	119 ^B^ ± 51	0.004
Urea (UREA)	mmol/L	7.4 ^A,a^ ± 0.3	7.0 ± 0.9	6.9 ± 0.9	6.4 ^a^ ± 0.8	4.8 ^A^ ± 1.4	0.001
Calcium (Ca)	mmol/L	3.1 ± 0.2	2.9 ± 0.4	2.9 ± 0.4	2.6 ± 0.5	2.9 ± 0.5	0.060
Phosphorus (P)	mmol/L	3.3 ^a^ ± 0.8	2.4 ^a^ ± 0.9	2.8 ± 1.1	3.9 ± 0.9	3.7 ± 1.1	0.007
Magnesium (Mg)	mmol/L	1.1 ^A,B^ ± 0.1	1.0 ± 0.1	1.1 ± 0.1	0.9 ^A^ ± 0.2	0.9 ^B^ ± 0.2	0.012
Iron (Fe)	μmol/L	24.7 ± 2.6	22.5 ± 2.8	27.8 ± 7.0	22.6 ± 10.9	27.6 ± 15.6	0.547

^1^ C—soybean meal; E1—5.0% pea seeds; E2—10.0% pea seeds; E3—15.0% pea seeds; E4—17.5% pea seeds. ^A,A^—values in the rows with the same letters differ highly significantly at *p* < 0.01. ^a,a^—values in the rows with the same letters differ significantly at *p* < 0.05.

**Table 5:** Biochemical and mineral blood indices ($\bar{x}\;\pm \;$SD), Experiment II.

Specification	Units	Groups ^1^					*p*-Value
		C	E1	E2	E3	E4	
Alkaline phosphatase (ALP)	U/L	119 ^A,B^ ± 16	111 ± 28	167 ^A^ ± 19	167 ^B^ ± 20	n.d.	0.001
Aspartate aminotransferase (ASPAT)	U/L	68.6 ^A,B,C,D^ ± 11.5	40.6 ^A^ ± 5.5	51.5 ^B^ ± 11.6	54.5 ^C^ ± 14.1	40.2 ^D^ ± 7.9	0.001
Alanine-aminotransferase (ALAT)	U/L	79.4 ^A,B,C,D^ ± 12.0	46.1 ^A^ ± 12.3	51.1 ^B^ ± 7.0	54.5 ^C^ ± 5.7	47.4 ^D^ ± 12.1	0.001
Albumin (ALB)	g/L	43.8 ^a,b^ ± 1.7	40.8 ^a^ ± 4.6	46.7 ± 2.0	43.2 ± 4.0	47.5 ^b^ ± 2.4	0.001
Total protein (TP)	g/L	79.7 ^A,B,C^ ± 4.6	67.6 ^A^ ± 10.3	66.2 ^B^ ± 8.6	59.7 ^C^ ± 5.5	78.2 ± 3.8	0.001
Glucose (GLU)	mmol/L	13.0 ^A,B^ ± 1.6	8.4 ^A^ ± 2.1	12.5 ± 2.7	15.0 ± 3.4	9.1 ^B^ ± 1.8	0.001
Cholesterol (CHOL)	mmol/L	3.0 ^A,a^ ± 0.2	2.5 ^A^ ± 0.5	3.1 ± 0.3	2.7 ^a^ ± 0.3	3.2 ± 0.4	0.001
Triglycerides (TG)	mmol/L	0.7 ^A,B,C,a^ ± 0.2	0.5 ^a^ ± 0.2	1.0 ^A^ ± 0.1	0.8 ^B^ ± 0.1	0.5 ^C^ ± 0.1	0.001
Creatinine (CREA)	μmol/L	185 ^A,b,a^ ± 11	166 ^a^ ± 20	102 ^A^ ± 16	120 ^B^ ± 23	185 ± 15	0.001
Urea (UREA)	mmol/L	8.2 ^A^ ± 1.0	7.6 ± 1.1	7.4 ± 0.9	6.3 ^A^ ± 0.9	8.9 ± 1.6	0.001
Calcium (Ca)	mmol/L	3.0 ^A^ ± 0.2	2.6 ^A^ ± 0.3	3.0 ± 0.2	2.9 ± 0.3	3.1 ± 0.2	0.001
Phosphorus (P)	mmol/L	3.7 ^A^ ± 0.6	3.4 ± 0.5	3.1 ^A^ ± 0.3	3.5 ± 0.5	3.8 ± 0.2	0.010
Magnesium (Mg)	mmol/L	1.2 ^A,B,C,D^ ± 0.2	0.9 ^A^ ± 0.1	0.9 ^B^ ± 0.1	0.9 ^C^ ± 0.1	1.0 ^D^ ± 0.2	0.010
Iron (Fe)	μmol/L	23.9 ^A^ ± 5.2	25.6 ± 4.6	27.4 ± 5.5	23.8 ± 3.5	39.2 ^A^ ± 4.5	0.001

^1^ C—soybean meal; E1—5.0% blue lupin seeds; E2—10.0% blue lupin seeds; E3—15.0% blue lupin seeds; E4—17.5% blue lupin seeds. ^A,A^—values in the rows with the same letters differ highly significantly at *p* < 0.01. ^a,a^—values in the rows with the same letters differ significantly at *p* < 0.05.
